# Associations between the built environment and obesity: an umbrella review

**DOI:** 10.1186/s12942-021-00260-6

**Published:** 2021-02-01

**Authors:** Thao Minh Lam, Ilonca Vaartjes, Diederick E. Grobbee, Derek Karssenberg, Jeroen Lakerveld

**Affiliations:** 1 Julius Center for Health Sciences and Primary Care, University Medical Center Utrecht & Utrecht University, Utrecht, the Netherlands; 2 Department of Physical Geography, Utrecht University, Utrecht, the Netherlands; 3 Global Geo Health Data Center, University Medical Center Utrecht & Utrecht University, Utrecht, the Netherlands; 4 Department of Epidemiology and Data Science, Amsterdam University Medical Centers (VUmc Location), De Boelelaan 1089a, 1081HV Amsterdam, the Netherlands; 5 Dutch Health Foundation, The Hague, the Netherlands; 6 Julius Global Health, University Medical Center Utrecht & Utrecht University, Utrecht, the Netherlands

**Keywords:** Obesity, Overweight, Built environment, Umbrella review, Food environment, Physical activity, Obesogenic environment

## Abstract

**Background:**

In the past two decades, the built environment emerged as a conceptually important determinant of obesity. As a result, an abundance of studies aiming to link environmental characteristics to weight-related outcomes have been published, and multiple reviews have attempted to summarise these studies under different scopes and domains. We set out to summarise the accumulated evidence across domains by conducting a review of systematic reviews on associations between any aspect of the built environment and overweight or obesity.

**Methods:**

Seven databases were searched for eligible publications from the year 2000 onwards. We included systematic literature reviews, meta-analyses and pooled analyses of observational studies in the form of cross-sectional, case–control, longitudinal cohort, ecological, descriptive, intervention studies and natural experiments. We assessed risk of bias and summarised results structured by built environmental themes such as food environment, physical activity environment, urban–rural disparity, socioeconomic status and air pollution.

**Results:**

From 1850 initial hits, 32 systematic reviews were included, most of which reported equivocal evidence for associations. For food- and physical activity environments, associations were generally very small or absent, although some characteristics within these domains were consistently associated with weight status such as fast-food exposure, urbanisation, land use mix and urban sprawl. Risks of bias were predominantly high.

**Conclusions:**

Thus far, while most studies have not been able to confirm the assumed influence of built environments on weight, there is evidence for some obesogenic environmental characteristics.

*Registration*: This umbrella review was registered on PROSPERO under ID CRD42019135857.

## Background

Obesity continues to be a major health issue and its wicked nature keeps challenging scientists and policymakers around the world [1]. In 2016, the World Health Organization (WHO) estimated that 1.9 billion adults, or 25% of the world’s population, are overweight; among which, a third is obese [2]. In 2015, high BMI contributed to four million deaths worldwide, 60% of which occurred in individuals with obesity and mostly due to cardiovascular diseases [3]. Between 1990 and 2015, the rate of early mortality due to high BMI increased from 41.9 to 53.7 per 100,000 individuals. Correspondingly, disability-adjusted life years due to high BMI increased from 1200 to 1630 per 100,000 individuals [3]. Given the striking worldwide prevalence of overweight and obesity and the resulting burden on individuals and societies, it is important to eludicate its determinants and find approaches for sustainable reduction and prevention**.**

Overweight and subesequently, obesity result from a chronic surplus in energy intake compared to energy expenditure, likely driven by an imbalance towards calorie consumption, sedentary behaviours and lack of physical activity [4, 5]. In the last two decades, there has been a paradigm shift in researching causes of obesity, in particular by moving the focus towards the drivers of such ‘obesogenic’ behaviours [6]. While earlier research generally focused on individual-level factors such as knowledge, psychological constructs such as motivation and also on genetics; more recent epidemiological research places obesity into the larger socio-ecological context where the environment also plays a role in shaping individual behaviours [1, 6, 7]. The built environment has been hypothesized to be a potential driver of obesogenic behaviours and ultimately, obesity [8–11]. Defined as all aspects of a person’s surroundings which are human-made or modified such as buildings, parks, facilities, and infrastructure; the built environment is a subset of the exposome, the totality of all exposures and lifestyle behaviours of an individual over a lifetime [12]. With the majority of the world’s population living in and spending most of their time in highly organized built environments, it is considered a relevant domain for epidemiological studies [13–15].

Frank and colleagues [16] conceptualizes the two main pathways where the built environment can contribute to health outcomes: one through behaviour and the other through direct exposure [16]. While the former refers to obesogenic behaviours such as physical activity and diet; the latter includes biological responses to environmental exposures, such as how air pollution might affect weight through inflammation. These two pathways are not mutually exclusive, increasing the complexity of built environmental studies. Nevertheless, with the research interest generated in the past two decades; various characteristics of the built environment have been extensively studied. Numerous primary studies on these characteristics have accumulated, which in turn produce a variety of systematic reviews, each with a specific range of included studies. For instance, some reviews focused explicitly on urbanization [17], greenspace [18] or walkability [19] whereas others combined primary studies that focused on a more diverse range of characteristics of the built environment [20–23]. As attention for the built environment continues to grow, we aim to gather the current state of evidence by systematically reviewing and reporting on published systematic reviews on the associations between the built environment and overweight or obesity.

This umbrella review examines the diverse built environment factors in broad strokes, thereby identifying crucial research gaps across disciplines as well as suggestions for future studies. Beyond the research sphere, this review enables policy makers, urban planners, public health workers and other professions at the intersection between the built environment and health to rapidly gain insights in the current evidence base in this field.

## Methods

Before the start of the search, this umbrella review was registered on PROSPERO under ID CRD42019135857. General reporting follows the guideline of Transparent Reporting of Systematic Reviews and Meta-analyses (PRISMA) and was reported in Additional file 1: Appendix 1 [24].

### Inclusion and exclusion criteria

We included systematic literature reviews, meta-analyses and pooled analyses of observational studies in the form of cross-sectional, case–control, longitudinal cohort and descriptive, ecological or intervention studies and natural experiments; from now on referred to simply as ‘reviews’ in this study, in general adult populations. Additional inclusion criteria were that they needed to: (1) report on at least one objectively measured built environment characteristic outside the home; (2) report on associations between these characteristics and weight-related outcomes in humans; (3) report on a systematic literature search, i.e. following a reproducible search strategy using a search string, and mention the databases in which the searches were done; (4) be published from 1 January 2000 onwards and be written in the English language. Reviews were excluded if they: (1) only focused on specific populations (e.g. people with obesity, pregnant women or athletes); (2) reported on unpublished materials such as conference abstracts, case reports, editorials and letters to editors; (3) reviewed studies on indoor home environments or other micro-environments; (4) had physical activity and/or dietary patterns but no weight-related outcome.

### Literature search

The search was conducted in May and June 2019 in seven databases: MEDLINE, EMBASE, CINAHL, Scopus, the Cochrane Database of Systematic Reviews (CDSR), the Joanna Biggs Institute (JBI) Database of Systematic Reviews and Implementation Reports, and the International Prospective Register of Systematic Reviews (PROSPERO). The search terms were built based on often used definitions and synomyms of the built environment and their commonly studied components, all possible operationalisations of weight-related outcomes such as weight status in categories, BMI, weight in kilograms, waist circumference, etc. and suitable geographical ranges. References of included studies were screened for any additional relevant studies that were missed. A detailed search strategy for each database can be found in Additional file 1: Appendix 2.

### Data collection and extraction

The literature search and removal of duplicates was done with support of a librarian. Then, two authors (TL and JL) screened titles and abstracts as well as full-text articles using Rayyan, a non-commercial web-based application [25]. Any disagreement in inclusion was resolved between these authors. Information extracted from reviews included:

- First author, year of publication, objectives of systematic review; eligibility criteria, study design and spatial coverage of primary studies such as countries, states, etc.
- Databases searched and temporal search range of each review.
- Built environment characteristics reviewed and where available, exposure methodology such as street audit, virtual audit, geographical modelling, etc.
- Weight-related outcome measures.
- General findings: quantitative results in case of meta-analyses in the forms of risk ratios, odd ratios or hazard ratios. Stratified results were extracted whenever available, otherwise, ratios of expected/ unexpected or positive/negative/non-significant associations where appropriate. In the case of descriptive reviews, main findings are summarized.
- Whether quality assessment tools were applied to included reviews.

### Methodological quality assessment

We asessed risk of bias utilizing ROBIS, a validated tool designed specifically to asses risk of bias in systematic reviews [26]. ROBIS include two main aspects, one is set out to identify concerns with the review process, and the other on judging risk of bias during different stages of the review including eligibility criteria and selection of articles, information collection, study appaisal and knowledge synthesis. There is also an optional first phase in assessing relevance of included reviews based on the PICO framework [27], the results of which are available upon request. The topical outcomes of ROBIS were presented in tabular and narrative forms. We also assessed the strength of evidence per domain of the built environment based on GRADE framework (Grading of Recommendations, Assessment, Development and Evaluation) [28].

## Results

### Literature search results

**Fig. 1:**
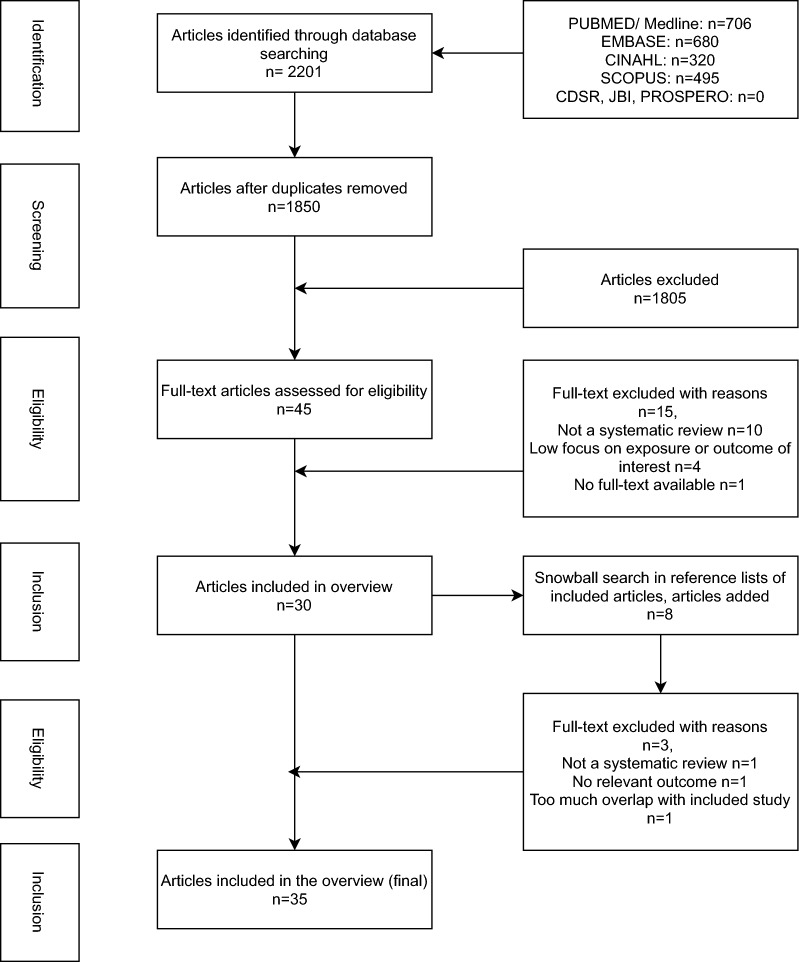
PRISMA flowchart of selected studies

The search identified 1850 unique articles from seven primary databases. Of these, 45 full-text articles were screened in which 30 met the inclusion criteria. Eight further articles were identified through a snowball search of the references and of these, six were excluded upon full text screening, resulting in a total of 32 systematic reviews that remained for synthesis. Figure 1 shows the PRISMA article selection process flow chart. Full references of included articles are provided in Additional file 1: Appendix 3.

### General overview of included reviews

#### Populations and designs

Key features of included studies are summarized in Table 1. About one third of the reviews (n = 12) examined general population while 10 reviews focused on a certain sub-population such as those from Western/developed countries, of which four explicitly studied North American populations. Four reviews focused on disadvantaged populations: including low SES, migrants, ethnic minorities or otherwise disadvantaged American communities (n = 3) or from developing/ low- and middle-income countries (LMICs) (n = 2). Overall, most retrieved reviews were conducted in developed or Western parts of the world (Table 1). Six reviews specifically included studies focusing on adults while the rest did not have age as an eligibility criterion.

**Table 1:** Summary of included studies

First author, number of included studies, year of publication, range of publication of primary studies in years	Study design and countries/populations covered	Exposure domain					Summary of results	ROBIS results
		Urban–rural	Food	Physical activity	Social inequality	Other		
Allender et al. [17], 9 studies, 2008, 1997–2007	All cross-sectional China, Russia, the Philippines, South Korea, South Africa, Cameroon, 1 global study	X					Low-strength evidence of the following findings: a positive relationship between BMI/ cholesterol and food share of household expenditure and proportion of population in urban areas; increasing urbanization improves micronutrients but increases body weight, blood pressure and cholesterol; lifetime exposure to urban environment is correlated with BMI; lifetime exposure to urban environment is correlated with obesity and there is rapid change in diet, obesity and physical activity among LMIC.	Unclear
An et al. [49], 16 studies, 2018, 2008–2017	Half longitudinal half cross-sectional US (9), Canada, Netherlands, Serbia, China, Italy, South Korea; adults study only in US, China, Italy and the Netherlands					Air pollution	Mixed associations, results vary by age sex and type of air pollutants. Associations between pollution and weight are probably not linear and mediated by health behaviours. Ratios of associations between exposure to respective pollutants and weight in adults by expected/unexpected/ non-significant: PM_10_: 5/0/1 PM_2.5_: 0/0/3 O_3_: 4/0/3 NO_2_: 3/1/3 SO_2_: 2/0/4 Overall: 1/2/6	Unclear
Angkurawaranon et al. [34], 45 studies, 2014, 1988–2013	All cross-sectional Malaysia, Laos, Vietnam, Thailand Indonesia, Timor-Leste, Philippines, Myanmar	X					Overall significant association between urbanicity and obesity. Pooled OR is 1.99 (95% CI: 1.64, 2.41) for South East Asia, in studies of only adults OR is 1.65 (95% CI: 1.36, 1.99) suggesting higher risks of obesity in urban areas. Heterogeneity in associations between and within countries, some can be attributed by economic status, age/sex, time of study, BMI classification used and populations studied. 35 studies have unclear risks, two studies have different response rate per area, the rest have low risks of bias.	Low
Black and Macinko [31], 36 studies, 2008, 1997–2006	STUDY DESIGN not clearly defined US (17), Sweden (1), Netherlands (2, Eindhoven), Scotland (2), Canada (1), Australia (1), multicounty (1)		X	X	X		PA environment is more consistently associated to obesity than the food environment Neighbourhood SES is consistently negatively associated with weight outcomes after adjusting for personal SES. Income inequality is positively associated to obesity but not on county level; racial composition studies have different results, but racial isolation is found to be associated with increased obesity prevalence.	Unclear
Casagrande et al. [50], 10 studies, 2009, 2002–2005	STUDY DESIGN not clearly reported All US studies; on county, multi-site, state- or nationwide samples			X			One study on the built environment and weight status, where higher BMI increased participants’ likelihood to report physical/ environmental barrier to exercising.	Unclear
Chandrabose et al. [29], 36 studies, 2019, 2007–2017	35 observational and 1 natural experiment, all longitudinal US, Canada, Sweden, Australia, Finland, Germany, UK (Wales), Lithuania			X			Evidence for association between walkability, recreational facilities (except greenspace), urban sprawl and obesity- presented by weighted z-score with positive, statistically significant results. Studies on stayers tend to have higher percentage of significant associations compared to movers. There was mismatch between perceived versus measured walkability in association with weight outcome. Methodological issues include residential self-selection and possible mediation by physical activity.	Low
Cobb et al. [21], 71 studies, 2015, 2005–2014	11 longitudinal, the rest cross-sectional US (64) and Canada (7)		X				Most of associations of food stores and weight are null, indices do have significant results more often but still dominated by nulls; quality of assessed studies sub-optimal- more than 30 studies have more than three errors. Ratios of associations between the following food stores with weight in adults by expected/unexpected/ non-significant: Supermarket: 4/22/67 Grocery store: 14/2/77 Fast food: 29/6/71 Relative healthiness food outlet index:16/1/20 (both adults and children) Combined healthy outlets: 2/0/2 Combined unhealthy outlets: 0/0/2	Unclear
Durand et al. [62], 44 studies, 2011, 2002–2009	Cross sectional (39) and longitudinal/ quasi-longitudinal (5) Sweden, US, Australia, UK, Belgium, Canada	X	X	X			Mostly non-significant associations between eight (over a total of ten) conceptual aspects of urban planning and weight. The other two aspects have no studies so far Associations in expected direction 0–33% of all studies, non-significant associations 66–100%.	High
Feng et al. [33], 63 studies, 2009, 2001–2008	61 cross-sectional, 4 longitudinal US (52), Australia (5), Canada (2), Denmark, Sweden, UK, European countries		X	X			Most consistent associations with sprawl index and land use mix, mixed results for fast food density. The overall ratio of expected/ unexpected/ non-significant associations is 40/2/38. There is difference in definition of “place” across studies, heterogeneity in metrics to measure the built environment and geographic range. There is added value in composite scores and indices compared to single measures in obesity studies; there is dominance of cross-sectional studies reduces causation or inference power.	Unclear
Ferdinand et al. [53], 169 studies, 2012, 1995–2010	164 quantitative observational and 5 qualitative observational; no specification whether cross-sectional or longitudinal Most studies within US (69), 60 outside and 40 with unknown locations		X	X			173/194 significant associations between food and PA environment with health benefits, results are similar in Southern states compared to others. Characteristics associated with higher scientific vigour have fewer significant associations with health benefits.	Unclear
Fleischghacker et al. [40], 40 studies, 2011, 1998–2008	Cross sectional except for Sturm and Datar (longitudinal, children) US, UK, Canada Australia, New Zealand		X				Six out of ten adult studies reported increased BMI with higher fast food access. Most studies do not have the same definition for fast food outlets or consistent sources of information or exposure methodologies. Fast food access is also associated to increased percent of minorities and lower SES	Unclear
Fraser et al. [38], 33 studies, 2010, 2002–2009	Cross-sectional and ecological, except for Sturm and Datar (longitudinal) US (12), Canada, UK, New Zealand (1), Australia (1)		X				Weak association between fast food access and obesity/ overweight. There are some associations between fast food and deprivation. Overall ratios of expected/ non-expected/ non-significant associations are 6/2/5; associations are significant especially when weights are self-reported. Highlighting inconsistencies in definition of fast food, various measures of access and geographical settings.	Unclear
Gamba et al. [39], 51 studies, 2015, 2004–2014	40 cross-sectional, 7 longitudinal, 4 repeated cross-sectional Only US		X				32% of all associations are significant in expected direction. Measure of presence of food stores is most likely to identify significant findings. Community nutrition environment should include more food store and food store types. Definition of fast food restaurants is not consistent across studies and quality of food data sources is sometimes questionable.	High
Giskes et al. [41], 28 studies, 2011, 2003–2009	Cross sectional except for 1 experiment (consumption) US, UK (obesity), Australia, Netherlands, Japan, New Zealand				X		Studies with weight-related outcome are mostly about access to/ density or presence of food sources; associations with weight as outcomes are notably more consistent than with diet. Ratios of associations in adults by expected/unexpected/ non-significant for weight status is 12:3:11 and for diet is 7:0:13.	Unclear
Grasser et al. [19], 34 studies, 2013, 1997–2010	33 cross-sectional, 1 prospective US, UK, Australia, Canada, EU			X			Walkability is more consistently associated with walking than weight outcomes; associations with weight outcomes were mixed and sometimes in the unexpected direction (especially for connectivity measure); some were significant in only a subpopulation (one city/ one gender etc.). Overall level of evidence was concluded to be low. In terms of quality: 8 good, 16 fair and 10 poor studies.	Unclear
Hernández et al [35], 18 studies, 2012, 1964–2010	14 cross-sectional, 1 retrospective 3 prospective cohorts Chile, Iran, Senegal, Kenya, China, Papua New Guinea, Peru, Panama, Guatemala, Tanzania, Poland, Bangladesh, India, Indonesia	X					Higher BMI in migrants compared to rural, lower BMI in migrants compared to urban, higher obesity in migrants compared to rural, lower obesity in migrant compared to urban in almost all measures of weight. Meta-analyses show no differences between urban and rural areas. Studies are highly heterogeneous. Same health profile observed for other cardiovascular conditions.	Low
Holsten [37], 7 studies, 2009, 2004–2006	6 studies were cross-sectional, 1 was ecologic All US except for Simmons (Australia)		X				Inconclusive results due to inconsistent correlations and various methods used. Most studies focus only on fast food restaurants and associations are only significant in one subset of populations.	High
Kondo et al. [47], 68 studies, 2018, 1991–2017	Experimental studies between (14) or within (21) subjects. 20 longitudinal, 3 case–control crossover, 9 quasi-experimental US (27), UK (13), Netherlands (5), Canada (5), Japan (4), Australia (3), Lithuania (3), Denmark (2), Germany (2), Finland, Italy and Spain (1 each)			X			Studies focusing on adult population found no association between BMI and green space exposure. There is a general lack of consistency in defining urban nature and measurement of its exposure hinders study quality and generalizability.	Unclear
Lachowycz et al. [18], 60 studies, 2011, 2002–2009	All cross-sectional Australia, Canada, England, Europe, New Zealand, Portugal, Sweden, Netherlands, US			X			Three studies with obesity-related outcome finds association between higher green space and lower adverse obesity-related outcome. Most studies found some sort of evidence for a relationship between greenspace and weight or reported mixed results across subgroups, according to measure of greenspace. Ratios of associations in adults by positive/ mixed/ negative/ non-significant is 3:6:1:4.	Unclear
Larson et al. [52], 54 studies, 2009, 1986–2008	Study type not mentioned in review, however discussion mentioned that they are mainly cross-sectional US only		X				Higher access to fresh supermarkets and limited access to corner stores are negatively associated with obesity while restaurant availability has mixed results. In general, more access to full restaurants & limited access to fast food restaurant is associated to less obesity. There is inequality in access to supermarkets, corner stores and restaurants (lower SES—more fast food, higher SES—healthier restaurants).	Unclear
Leal and Chaix [32], 131 studies, 2011, 1985–2009	14 longitudinal (9 on weight), the rest cross sectional US (86), Sweden, UK, Canada, Netherlands, Germany & Czech Republic, France, Italy, Lithuania, Portugal, Slovakia, Switzerland, Australia, Japan, New Zealand, Spain	X	X		X		Most consistent association is between low SES and increased weight; low urbanized associated with high weight; high supermarket low convenience store low fast food associated with low weight; high street connectivity, high density of intersections and services associated with lower obesity; criminality and insecurity associated with high weight. High traffic noise associated with high triglyceride level.	Unclear
Lovasi et al. [51], 45 studies, 2009, 1995–2009	Study type not mentioned in review, either individually or in statistics summary All US		X	X	X		Targeted groups disadvantaged in terms of access to food stores, fast food outlets, places to exercise, aesthetics and safety. Strongest support for importance of food stores, exercise facilities and safety. The built environment might affect the high SES group more than the low SES groups due to low exposure. Built environment interventions are more effective if targeted to the disadvantaged; especially those that help reduce disparities.	Unclear
Mackenbach et al. [23], 92 studies, 2014 2003–2013	6 longitudinal, 84 cross-sectional, 2 both US (74), Canada, Australia, New Zealand, UK, Belgium, Portugal, EU, France, Denmark		X	X			Heterogeneity in metrics used and findings, except for urban sprawl and land use mix with clear associations with obesity within North America. Stratification results show remaining heterogeneity within continents, mode of measurement, methodological quality. Overall weak associations between environment and weight status. 29 strong, 53 moderate and 8 weak primary studies methodologically. Reporting was moderate or strong in quality.	Low
Malambo et al. [45], 18 studies, 2016, 2005–2015	17 cross-sectional and 1 longitudinal (study on stroke) US, New Zealand, Australia, China, Sweden, Canada		X	X			BMI is lower in walkable neighbourhoods with recreationally dense neighbourhood. BMI is higher in places with high densities of fast food restaurants not supermarkets (both American studies in somewhat older populations).	High
McCormack et al. [43], 55 studies, 2019, 1998–2017	Cross-sectional (36), prospective and retrospective cohort, longitudinal, case–control, case-crossover, time series, quasi-experimental Canadian provinces Ontario, BC, Quebec, Alberta, Nova Scotia		X	X			Consistent associations between aggregate built environment score, greenness, land use, food environment and weight status. Somewhat less consistent associations for population/ dwelling density and mostly non-significant for route characteristics.	High
Papas et al. [20], 20 studies, 2007, 2002–2006	18 cross-sectional investigations, three of which were ecologic studies. Two longitudinal studies US, Australia, and Europe		X	X			Food environment is less well studied than PA environment, associations differ by age groups or races. 17/20 studies found statistically significant associations between aspects of the built environment and weight. Concerns include inconsistency of measurements of the built environment across studies, the cross-sectional design of most investigations, and the focus on aspects of either diet or physical activity but not both.	Unclear
Patterson et al. [48], 10 studies, 2019, 2008–2017	Longitudinal, controlled trials or natural/ quasi experiments US (6), UK and China			X			Meta-analysis of five studies show initiating public transport use was associated with 0.30 units BMI reduction 95% CI (0.14, 0.47). Distance from the residential address to the nearest bus route had mixed associations with adiposity.	Low
Renalds et al. [46], 23 studies, 2010, 2005–2008	Mostly cross-sectional, 1 experiment, 1 retrospective review of longitudinal study Countries not listed but seem mostly US		X	X			Mixed land use alone is not sufficient for BMI research, more investigation into specific land use (type of business within a residential neighbourhood) is needed. Urbanity, low land use mix, crime, low street connectivity, automobile dependency increases overweight in adults.	High
Schüle and Bolte [30], 33 studies, 2015, 2005–2013	Most cross-sectional except for one British study Belgium, Australia, Canada, US, Sweden, Germany, UK		X	X	X		Nine studies looked at adult obesity and/or BMI. Eight out of 11 studies had significant associations between neighbourhood SES (NSES) and BMI/ overweight/ obesity: four high SES—low BMI and four low SES—low BMI; seven studies found significant association between built environment and BMI independent of NSES. Two studies found associations between NSES and individual characteristics (sex/ race); six studies found interaction between BE and individual SES (ISES, in other words, associations are only significant for some subgroups: women, whites, car owners etc.) Differences between NSES and ISES should be considered: it is not clear how NSES, built environment and sex interacted with one another.	High
Sugiyama et al. [22], 41 studies, 2014, 2010—2013	34/41 cross-sectional, 6 prospective, 1 both US (23), Canada, UK, Australia, France, Brazil, Egypt, Belgium & Nigeria			X			Composite environmental indices have significant associations with weight related outcome (sprawl index, pedestrian + public transport + residential density) unless it is walkability. Among the walkability components, land use mix is most consistently correlated to weight outcomes. Utilitarian destinations also have significant associations. Leisure-activity related attributes do not contribute to obesity/ weight. Transport-related physical activity should be a target for future studies. Ratios of associations in adults by expected: unexpected: non-significant in cross-sectional studies is 56/6/86 and in prospective studies is 9/1/10.	Unclear
Tseng et al. [42], 17 studies, 2018, 2008—2018	17 natural experiments UK, Australia but mostly US (12)		X	X			Low level of evidence for weight/ BMI change in intervention groups. Four out of nine interventions in PA has significant BMI reduction (ratio reduction/ inconsistent/ no difference is 4/3/2). None of the five food interventions shows BMI reduction. The mixed environment studies (both food and PA) also have low level of evidence. Studies with significant results show associations with low clinical significance. Most studies have high risk of biases.	Low
Wilkins et al. [36], 113 studies, 2019, 2005–2018	87 cross-sectional, 26 longitudinal US, UK, Australia, Germany, New Zealand		X	X			Overall, null associations dominated other associations. Fast food outlets associations more positive when defined more narrowly, measured in proximity rather than presence, and in low SES groups. Percentage of associations in adults by expected/unexpected/ non-significant for each outlet is: Fast food: 20.8/4.2/75.0 (404 associations in total) Convenience stores: 10.9/8.5/79.6 Supermarkets/groceries store: 6.6/12.9/80.5 Restaurants: 6.3/18.4/75.3 Diverse methods used to measure food environment in five different aspects: exposure data source, extraction method, methods and definitions of food outlets, geocoding method and retail food environment metrics. Measurement methods are not well reported.	Low

*BMI* body mass index, *LMIC* low and middle-income countries, *SES* socioeconomic status, *OR* odd ratio, *CI* confidence interval, *PA* physical activity, *BE* built environment, *US* United States of America, *UK* United Kingdom. For ROBIS result, the final judgment presented here as “low”, “high”, “unclear” risk was reached by answering a string of questions regarding multiple aspects of the original review. Unclear risks mean that there was insufficient information reported to judge whether risk of bias was high or low

Most reviews found more studies with a cross-sectional design than longitudinal; apart from Chandrabose et al. [29] who only included longitudinal studies by design [29]. The number of primary studies included in the reviews ranged from seven to 169. The number of databases searched varied from one (n = 3, PubMed or Medline) to five or more (n = 8) up to a maximum number of 13 databases. Most studies (n = 25) deployed a hand search and/or snowball additionally to database search, often to search grey literature and non-academic sources for articles. The primary studies in included reviews were published between 1964 and 2018.

#### Exposures

Ten reviews examined a variety of factors of the built environment; five focused on environmental factors related to physical activity including walkability (*n* = 1), greenness (*n* = 2) or transport (*n* = 1); eight on the food environment, three on urban- rural difference and urbanization and five on social disadvantages; and finally, one review examined air pollution. We did not identify any eligible reviews on conceptual aspects of the built environment such as sports facilities or motorized transport. Whenever reported by the authors, we counted the percentage of findings in the expected or theorized directions (Table 2). This figure ranged from 11 to 89% among the 15 eligible reviews.

**Table 2:** Summary of quantitative results in selected reviews

Study^#^	First author	No. of primary studies	% longitudinal	No. of continents covered	No. of countries covered	Overall quality	Domain					% of findings in expected direction
							Urbanicity	Food	PA	Socio-econ	Pollution	
1	An	16	50	3	4	2	0	0	0	0	1	11
2	Cobb	71	15	1	2	2	0	1	0	0	0	19
3	Durand	44	11	3	6	3	1	1	1	0	0	33
4	Feng	63	6	3	7	2	0	1	1	0	0	50
5	Ferdinand	169	N/A	N/A	N/A	2	0	1	1	0	0	89
6	Fleischghacker	40	3	3	5	2	0	1	0	0	0	60
7	Fraser	33	3	3	5	2	0	1	0	0	0	46
8	Gamba	51	14	1	1	3	0	1	0	0	0	32
9	Giskes	28	0	4	6	2	0	0	0	1	0	46
10	Lachowycz	60	0	3	9	2	0	0	1	0	0	21
11	Papas	20	10	3	3	2	0	1	1	0	0	85
12	Schule and Bolte	33	3	3	7	3	0	0	0	1	0	36
13	Sugiyama	41	17	4	9	2	0	0	1	0	0	39
14	Tseng	17	0	3	3	1	0	1	1	0	0	44
15	Wilkins	113	20	3	5	1	0	1	1	0	0	11

^#^No. of studies: number of primary studies included, % longitudinal: percentage of primary studies that were longitudinal, no. of continents: number of continents present in the review, no. of countries: number of countries present in the review, overall quality: ROBIS results of the review as assessed in our umbrella review, domains depicted the different domains that were covered in the review and % expected associations is the percentage of associations in the review that was in the expected/ theorized direction. Domain names: *PA* physical activity, *socioecon* social inequality

#### Outcomes

The range of weight-related outcomes examined included individual (change in) weight, weight status, BMI, waist circumference, body fat percentage, weight to height ratio, skinfold measure, weight-to-hip ratio and population measures such as odds of being overweight and obese, or prevalence of obesity. Individual weight-related outcomes were both self-reported and/or objectively measured. Some reviews also focused on exploring confounders and modifiers of the relationships between built environment and obesity, such as individual or neighbourhood socioeconomic status (SES) [30, 31]. Nine studies examined also other health outcomes such as cardiometabolic or cardiovascular conditions besides behavioural and weight-related outcomes.

### Quality assessment

Using the ROBIS tool, we identified seven reviews to have low risk of bias, 18 with unclear (or medium) and seven with high risks of bias. Most common quality-lowering traits of the included reviews were lack of a review protocol, lack of quality assessment of primary studies, and time and language restrictions on literature search (see also Table 1 and Additional file 1: Appendix 5). The domain-wide evidence levels ranged from very low (for air pollution and the food environment) to low (physical activity environment and urbanicity). The highest evidence level was moderate for social inequality (Table 3).

**Table 3:** Summary of evidence for associations between domains of the built environment and weight outcome

Domain	Associations with weight (if domain is higher)	Strength of evidence*
Urbanicity	Lower weight in HIC and higher weight in LIMC	Low
Food environment	Overall null	Very low
Fastfood	Some evidence for higher weight	Low
Physical activity environment	Overall inconsistent	Low
Walkability	Lower weight	Low
Greenness	Lower weight	Low
Transport	Inconsistent	Very low
Sports facilities	No systematic reviews found	–
Motorised transport	No systematic reviews found	–
Social inequality	Confounder at both individual and neighbourhood level	Moderate
Air pollution	Inconsistent	Very low

^*^Strength of evidence is composed based on GRADE framework (Grading of Recommendations, Assessment, Development and Evaluation) [67]

### Thematic results

#### Urban—rural differences

Overall, there is evidence for disparity in overweight and obesity between urban and rural areas (Table 3), although the direction of associations is not homogenous. In their review, Leal and Chaix (2011) found that residing in urbanized neighbourhoods or neighbourhoods with higher residential density in developed countries was associated with lower weight [32]. Other reviews, which were also conducted in developed countries, found that urban sprawl, a feature of the urbanization process usually operationalized by population density, mixed land use, intersection density, block size and street accessibility was positively associated with obesity (i.e. more sprawl, less urbanised, more obesity) [23, 33].

Three other reviews in developing countries also found associations between urbanicity and obesity, however the direction of association was reverse; such that more urbanized areas had relatively more overweight and/or obese populations [17, 34, 35]. A narrative study on urbanization by Allender et al. (2008) found that increasing urbanization improved food micronutrient contents of diet but also increased body weight, blood pressure and cholesterol in developing countries [17]. Lifetime exposure to an urban environment was positively correlated with BMI and obesity, even though the strength of associations was low [17]. Angkurawaranon et al. (2014) conducted a review specifically for South East Asia and found heterogeneity in associations between urbanity and weight-related outcome both between and within countries; which could be attributed partially to relative economic status, age and sex [34]. A meta-analysis included in the same study showed an overall OR for being overweight of 1.65 (95% CI: 1.36, 1.99) for adults who lived in urban areas versus rural [34]. Hernández et al. (2012) reviewed literature on rural-to-urban internal migration in eight developing countries and observed that both BMI and obesity rates generally increased in those who migrated from rural to more urbanised areas [35]. Migrants’ BMIs were 0.2–3.8 kg/m^2^ higher than rural counterparts and 0.3–1.3 kg/m^2^ lower than urban counterparts [35]. The study also reported differences in other weight-related anthropometric measures such as waist circumference, hip circumferences, and triceps skinfold, which were assessed in only a few primary studies. Overall, all three reviews acknowledged a change in diet, obesity and physical activity pattern towards sedentary over time among all LMICs studied. However, they did not sufficiently explain the rural urban disparity in weight and BMI and the difference in trend between developed and developing world.

#### Food environment

Earlier reviews from the 2000s observed that more studies were done on physical activity environments than on the food environment in relation to obesity [20, 33]. Ever since, more research and systematic reviews on characteristics of the food environment emerged, and in our umbrella review we found eight reviews that focused primarily on the food environment in relation to weight status outcomes. For this domain, null associations dominate the results [21, 36–38]. The latest and most comprehensive food environment study by Wilkins et al. (2019) found 70.3 to 77.7% of the examined associations between different food outlets and adult obesity to be non-significant [36]. While the figure is slightly lower for American studies at 50–59% [39] the inconsistency in associations across reviews is comparable. Most reviews attributed this inconsistency to a large heterogeneity and inconsistency in defining the food environments under study, as well as the components and methodologies to measure them [21, 36–40]. Even within the fast food domain where associations were most consistent, there was much heterogeneity in what was regarded as fast food retail, for example, whether only the large fast food chains were qualified or also small corner stores [38, 40]. Notably, Wilkins et al. (2019) further concluded in a recent review that a narrower definition of fast food led to more positive associations [36]. On the other hand, Cobb et al. (2015) found that composite food outlet measures which combine both healthy and unhealthy food outlets were more consistently associated with weight in adults than measures of single food outlet types [21]. Interestingly, Giskes et al*.* (2011) found that associations between access to and density of food outlets were more consistent in their relation to weight than to dietary behaviours, suggesting a gap in understanding between behavioural and physiological outcomes [21]. Wilkins et al. (2019) further assessed quality of reporting in food environment studies and concluded that most exposure methodology sections did not meet their newly developed GeoFERN framework [36], while Cobb et al. (2015) lamented the low quality of primary studies [21]. Another aspect frequently discussed was the quality of data on food outlets. Some used indirect measures and sometimes outdated data, despite the fast development of the foodscape [39].

In reviews that included food among other components of the built environment, mixed results were also observed. Tseng et al. (2018) found no change in BMI in any intervention studies regarding the food environment. Interventions targeting both food and physical activity environment also did not result in BMI change, though most primary studies suffered from high risks of bias [42]. McCormack et al. (2019) however found significant associations in examined observational studies that investigated Canadian food environments and weight status [43].

#### Physical Activity environment

##### Walkability

Walkability in epidemiological terms is an index of environment characters that are conceptually associated with active transport such as walking or cycling [44]. To calculate walkability, a multitude of components are combined, among which are residential density, land use mix, destination facilities, street connectivity measured by intersection density, and sometimes route characteristics (e.g., greenspace). Walkability indices have mixed associations with weight. Grasser et al. (2013) found the walkability index to be consistently positively associated to walking and to some extent, cycling behaviours but much less to weight outcomes [19]. Three general reviews including one meta-analysis [29], one Canadian review [43] and Malambo et al. 2016 [45] found more consistent evidence for associations between neighbourhoods with high walkability scores and lower BMI; although some mismatch was reported between perceived and objectively measured walkability. It was furthermore indicated that the role of physical activity as a mediator should be further explored [45].

Many reviews also examined the associations between individual components of walkability and their associations with weight. Some components are found to be more consistently associated with weight while others have mixed results, similar to the index they represent. Density measures such as population density, housing unit density or address density had equivocal associations with weight-related outcomes [19, 33]. Sugiyama et al. (2014) found that walkability as a composite score was less correlated to weight than land use mix [22]. This view was also supported by two literature reviews which examined a broad variety of built environmental factors in associations with weight and weight-related outcomes in developed countries [23, 33]. Both reviews found land use mix (together with urban sprawl) to be most consistently associated with weight. An earlier review by Renalds et al. (2010) also highlighted that land use mix was a consistent factor, but pointed out that more studies should examine specific land uses that are relevant to weight change, in order to elucidate the mechanistic pathways of this association [46].

Green space was studied both as a separate environmental factor relevant to weight but also as a component of walkability. Two included reviews focus on green space as the primary exposure: Lachowycz and Jones (2011) specifically studied access to green space and obesity and found some evidence for an association but generally results were mixed, depending on different weight-related outcomes under study [18]; Kondo et al. (2018) reviewed broader health outcomes and found no association between green space and weight in three primary studies in adults [47]. In reviews that focused on green space next to other environmental aspects, McCormack et al. (2019) found consistent associations between greenspace and weight status in Canadian settings [43] while meta-analysis results by Chandrabose et al. (2019) observed no significance in associations [29]. Other components of walkability such as route characteristics, street connectivity, and facilities at destination had non-significant associations with obesity [19, 29, 32, 43, 45, 46].

##### Public transport

Except for walking and cycling, other means of transportation have been relatively understudied in obesity reviews. An early review by Renalds et al. (2010) found that residents of urban settings with high commuting times and sparse public transport networks had a higher likelihood of being obese [46]. A recent meta-analysis by Patterson et al.(2019) indicated that switching from personal to public transportation through new infrastructure or by adjusted ticket pricing was associated with 0.3 (95% CI: 0.14, 0.47) kg/m^2^ reduction in BMI [48]. Interestingly, Tseng et al. (2018) found that while only less than half of the environmental policy interventions produced statistically significant associations with weight; these associations were still more consistent than for diet or physical activity outcomes [42]. Across all interventions, those related to public transports had the most significant associations, even though the included primary studies overlapped largely with Patterson and colleagues [48]. For other transport measures including distance to the nearest bus route there was limited and inconsistent evidence.

#### Air pollution

An et al. (2018) reviewed air pollution studies in relation to weight status [49]. Most air pollutants studied came from urban anthropogenic activities such as fossil fuel burning for heating and transportation. The primary studies in this review were highly heterogeneous in pollution measurement methods and associations varied by age, sex and type of pollutants. However, in the included studies of adults, weight status was only consistently and adversely associated with particulate matter below 10 µm in diameter (PM_10_).

#### Social inequality

Although socioeconomic factors are not considered physical aspects of the built environment, their important conceptual role in influencing both urban design, environmental exposure and weight-related outcome is worth examining. Four reviews on social inequality in relation to environments and weight status were conducted in the United States. Casagrande et al. (2009) and Lovasi et al. (2009) found that some ethnic minority groups such as African Americans and Mexicans were more disadvantaged in terms of access to healthy food options, exercise facilities and outdoor environmental safety [50, 51]. Larson et al. (2009) reported inequality in access to food sources, especially individuals with a lower SES were more exposed to fast food while the higher SES groups had higher access to healthier restaurants [52]. On top of that, Lovasi et al. (2009) suggested that the built environmental characteristics affected higher SES group disproportionately because the lower SES groups were found to have much lower exposure to any food outlet in general [51]. One review that stratified studies from the Southern states found no difference in beneficial effects of the built environment on weight compared to the rest of the studies [53].

Reviews conducted in general populations found similar patterns. Black and Macinko (2008) and Renalds et al. (2010) found income inequality, crime and racial isolation to be associated with obesity in developed countries [31, 46]. Allender et al. (2008) found that BMI and cholesterol levels were positively correlated with food share of household expenditures in LMICs, though this evidence is based on one single primary study [17]. Three other studies reported association between fast food exposure and increasing deprivation, low SES or ethnic minority [36, 38, 40]. Taken together, all these reviews suggest the emerging role of social inequality both as confounder and independent component of the built environment relevant for weight.

## Discussion

Our umbrella review identified 32 systematic reviews examining associations between aspects of the built environment and weight-related outcomes. About a third of the reviews focused on general built environmental characteristics while others focused on specific aspects such as the food environment, physical activity environment such as walkability and active transport, urbanization, or neighbourhood SES. Overall, the results indicate that associations were very small or absent for the physical activity environment and the food environment, however the included reviews found comparatively more consistent associations for the physical activity environment compared to the food environment, especially in North American settings [31, 50]. Generally, measures of fast-food exposure, urban sprawl and land use mix are most consistently associated with weight outcomes. In the relation between the built environment and weight, we found evidence for the role of social inequality both as confounder for associations between the physical built environmental factors and obesity, as well as being independently associated to obesity itself.

One might argue that the association between the built environment and weight is a bridge too far, and that it would be logical to review the epidemiological evidence on associations with intermediate, more proximal outcomes, such as obesogenic behaviours [54]. These behaviours are conceptualized mainly through energy imbalance: higher obesogenicity means a surplus of energy intake and a deficiency of energy expenditure. In terms of energy intake, Sleddens et al. [55] carried out an umbrella review for environmental correlates of dietary behaviours and found mixed results for the built environment. Evidence was suggestive at best and even so, correlations were stronger for personal behaviours such as habit, self-regulation, self-efficacy and motivation compared to elements in the built environment. In terms of energy expenditure, an umbrella review was carried out in 2017 for correlates of physical activities, where most of the associations for specific behaviours such as walking, cycling and active transport were shown to be inconsistent [56]. The most consistent association was found between neighbourhood walkability, street connectivity, land use mix, level of urbanity and overall physical activity level. These studies mirror our review results for the food and physical activity environments, respectively.

One of the probable causes of inconsistency in association direction is the variety in definitions used for environmental exposures. The definitions of urbanity or urbanization [17, 47], food in general and fast food in particular [40] and components of walkability differed greatly between primary studies [19]. Feng et al. [33] also discussed the various geographical definitions of place or area under study, and whether neighbourhood, city or county should be the most appropriate level of analysis. Unsynchronized definitions, together with diverse operationalisations such as: access to, accessibility of, or relative density of built environmental factors have led to a myriad of environmental measures used between studies [33]. As of now, the field is still developing and we seem far from adopting a gold standard or best practice in built environment exposure assessment and operationalisation. On the other hand, one might argue that single gold standards should not be strived for, as the operationalisation of the ‘best’ exposure measures for many environmental characteristics are likely to be context-dependent and will vary from place to place, and from population to population. However, universal guidelines on transparent and complete reporting of methods and findings in environmental epidemiological studies will facilitate interpretation of, and comparison across, such studies [57].

In terms of effect sizes, the evidence found so far indicates three main issues: (1) effects are likely to be small (2) there is space for improvement of epidemiological studies regarding the built environment in general and, more importantly, (3) our conceptual understanding of the ecological relationship between the built environment and personal behaviours and consequent weight may not be optimal. Even though it is out of the scope of our study to examine subjective assessment of the built environment (i.e. self-perception), some studies suggest that their role in modifying health behaviours should not be underestimated [54]. Moreover, it is increasingly being acknowledged that the interactions between humans and their environments take place in a complex system that continuously interacts and adapts, and where the built environment is only one of the many nodes in this interactive network [58]. Obesogenic behaviours do not occur in isolation but are a product of myriad of individual-level and contextual processes. Therefore, the picture of built environments and weight is framed within a larger context of interrelated human–environment interactions, which also include factors from the sociocultural-, economic-, and policy environments [8]. Even when the goal is to capture a snapshot, we should not lose sight of this big picture.

Recently, researchers started to study the combined influence of environmental factors, rather than focusing on traditional single-exposure single-outcome epidemiological approaches, in order to attempt combining these snapshots together to make sense of the big picture. For example, in the included reviews, indices including multiple food outlets were more consistently associated with adult obesity compared to individual outlets [21]. Moreover, Cobb and colleagues, as well as other authors highlighted the need to combine built environmental factors for two reasons: (1) their effect might not easily be singled out on individual exposure basis and (2) people are exposed to multiple built environmental factors at the same time anyway [22, 33]. This methodology does not come without challenges, one being that the combining of environmental factors (for instance in an index), much like the rest of exposure assessment, is not standardized. Even though guidance is available, such as one offered by Organisation for Economic Co-operation Development [59], some steps of the process (such as weighting the components) are subjective and entirely at the authors’ discretion. Another issue is the often-challenging interpretability of the product score, therefore the process of index making must be transparently reported.

Twenty-five out of thirty-two included reviews had considerable risks of bias, mostly because there was no reported protocol for review and assessment of quality of primary studies. To some extent, this limited the validity of the synthesized evidence and therefore lowers the overall rigor of the current review. Included reviews tended to use pre-set protocols only from 2011 onwards. On a related note, no two systematic reviews in the current umbrella review used the same quality assessment tool. It has also been acknowledged by other reviews that quality assessment is yet to be standardised [60]. Moreover, there is also discrepancy on how the authors processed information from quality assessment: some stratified their analysis based on quality of studies, either in meta-analyses or descriptive analyses, but most studies simply reported the quality in their discussion.

Except for one review that explicitly studied confounding effects of individual- and neighbourhood SES [30], no other review took confounders into account when assessing the strength of associations between the built environment and weight. The modifying role of age and sex were occasionally explored, and results were sometimes stratified, but they were rarely discussed. Some built environmental exposure was found to be related to obesity especially for some subgroups such as women, whites or car owners. Such interesting results should be further discussed in future studies, as has also been indicated by others [19, 37, 53]. Furthermore, some studies found that characteristics in the built environment and BMI were associated independent of or dependent on individual SES. Black and Macinko (2008) and Schüle and Bolte (2015) examined both individual and neighbourhood SES both as independent predictors and as mediators for associations between built environment and obesity [30, 31]. Black and Macinko (2008) found neighbourhood SES to be consistently associated with weight, even after adjusting for individual SES, while Schüle and Bolte (2015) found significant associations between neighbourhood SES and BMI in both directions, suggesting that SES might be an independent factor next to their suggested role as confounders for many environment exposures.

Ding and Gebel carried out an umbrella review on the built environment, physical activity and obesity in 2012 [61] in which six included reviews overlap with our umbrella review [18, 20, 31, 50, 51, 62]. Compared to the evidence base at that time as reported in their review, there have been positive developments: environmental factors and weight associations have increasingly been based on conceptual guidance; the importance of socioeconomic status continued to be stressed in built environment studies and was adjusted for in many recent studies [61]. However, some issues in primary studies remained poignant: despite improved methodological rigour of exposure assessment, these efforts have not borne fruit, neither in increased significant associations nor in more consistency in association directions, and measures used remained heterogeneous. Most recent reviews, including this study, still find that cross-sectional studies dominate the research landscape, which limits causal inference of the studied associations. In addition, most primary studies still take place in the Western world while obesity incidence in LMICs is still rising compared to high-income countries. Quality of reporting, especially that of methodologies, has been improved but is still considered to be insufficient [36].

This umbrella review offers a comprehensive overview on the evidence of different aspects of the built environment pertaining to weight and weight-related outcomes. The strengths are that we were able to consolidate many different aspects of the built environment over time, and that we included reviews from a variety of disciplines such as epidemiology, urban planning, social sciences, and public health. Moreover, by carefully examining the quality of the included reviews, we were able to identify crucial issues with past and current systematic reviews on the built environment, which have not been done before, while keeping a close eye for possible improvements in both systematic reviews and primary studies.

Limitations of this umbrella review include the possibility of missing recent primary studies in relatively understudied fields since we only included systematic reviews. This might be a weakness in a fast-developing field such as environmental epidemiology. For example, we had to exclude motorized transport due to the lack of systematic reviews even though other forms of reviews for this topic already exist [63] and there was no review on sports and recreation environment even though primary literature was present. A recent Nature study suggested that the gap of BMI between residents in urban and rural areas is closing, mostly by an unprecedented increase in rural BMI across the globe in recent years [64]. Moreover, some included reviews, especially the earlier ones, tend to have high overlap in primary studies, which might overestimate the strength of evidence for some aspects of the built environment.

To move forward, future studies must address many challenging issues regarding exposure assessment as well as the operationalisation of exposure variables, and in the analyses take into account the complexity of real life. The longitudinal design of many cohorts can be used to increase causal inference power of environmental correlates. This requires exposure data themselves to be routinely collected and updated in time to make these analyses possible. In terms of exposure, some aspects of the built environment need further exploration as pointed out by Durandet al. (2011), who found the community aspect of the smart growth principles; which also include walkability, mixed land use, public transportation and compact building among others; to be missing in health analyses [62]. Studies incorporating social networks and the spread of obesity are developing, but require complex modelling and therefore are mostly still in its infancy [65, 66]. Other understudied principles include predictable, fair and cost-effective policymaking which is more abstract but not less important for future studies. Moreover, less recognised obesity-related exposures such as air pollution should be further studied. Thus far, there is limited evidence for this domain of exposure, as this topic in adults came from one single primary study [49]. Furthermore, some reviews on specific topics such as green space might have become outdated and this needs an update. In a broader stroke, future studies could make use of increasingly enriched open environmental data to explore novel factors of the built environment relevant to health. To this end, relevant policies should be in place to encourage data sharing between stakeholders, such as the European Union’s INSPIRE Directive^1^ or the American Open, Public, Electronic and Necessary (OPEN) Government Data Act.^2^ In addition, BMI as a proxy of overweight and obesity is simple to measure but is not sufficient to define central obesity. Incorporation of measures such as body fat percentage or waist circumference should become standard practice in the future.

In terms of analysis, a recurring theme in many included reviews is the suggestion to incorporate complexity into current epidemiological studies. This improvement is multi-faceted: on the one hand, we have mentioned earlier in this review the use of indices to better quantify simultaneous exposure. On the other hand, non-linear complexity could be incorporated in statistical analysis by moving beyond the reductionist linear modelling method, especially for an outcome such as weight where either extreme is considered adverse [6, 58]. Fortunately, there has been a recent recognition that the low effect size in the current environmental epidemiological studies might be attributed to the isolated single-exposure single-outcome and linear modelling method, neither of which is realistic in terms of human interactions with the environment [67, 68]. Traditional epidemiology however could already benefit from bolder exploration of mediation and interaction effects of dietary or physical activity behaviours to further strengthen the causal inferences to the current associations [54, 69]. Moving forward, innovative methodologies such as agent-based modelling and other self-learning algorithms could be used to improve our understanding, by allowing interactions in the forms of various simulated scenarios in environmental changes and their consequences on weight [70].

As for future systematic reviews, included reviews suggest that both individual and neighbourhood SES are important effect modifiers for the associations between the built environment on health and thus both should be considered in future studies; especially in terms of possible interactions between neighbourhood SES, built environment and sex or age [30]. Finally, as the number of reviews continue to increase, it is recommended that future reviews in the built environment follows PRISMA reporting guideline and pre-register in a dedicated registry such as PROSPERO or HRB Open Research to ensure transparency and prevent overlapping in review topics. The use of quality assessment should be taken seriously with standardized, validated tools suitable for each study design, as recommended by trusted sources such as the Cochrane Center.

In conclusion, while evidence for associations between most built environmental characteristics and weight related outcomes were null or equivocal, some characteristics have a more consistent link, such as fast-food retail exposure, urbanisation, land use mix and urban sprawl. Risks of bias was predominantly high, and we pointed out aspects in the methods, measures, analyses, and reporting that may increase our understanding of the assumed influence of built environments on obesity in future studies.

## Supplementary Information

**Additional file 1: Appendix 1.** PRISMA checklist for reporting. **Appendix 2.** Full search strategy for all databases. **Appendix 3.** Full references of included reviews. **Appendix 4.** Risk of bias assessment of included studies using ROBIS tool.

## Data Availability

Not applicable.

## Footnotes

1 Information on the directive can be found via the European Commission’s website https://inspire.ec.europa.eu/inspire-directive/2.

2 Information on the OPEN Government Data Act can be found on Data Coalition’s website https://www.datacoalition.org/policy-issues/open-data/open-government-data-act/.
