# Supplementary material for: Associations between the built environment and obesity: an umbrella review
Source: Int J Health Geogr. 2021 Feb 1;20:7. doi: 10.1186/s12942-021-00260-6 (PMC7852132; doi:10.1186/s12942-021-00260-6)
Supplement: Supplementary file 1 — Additional file 1: Appendix 1. PRISMA checklist for reporting. Appendix 2. Full search strategy for all databases. Appendix 3. Full references of included reviews. Appendix 4. Risk of bias assessment of included studies using ROBIS tool. [file 12942_2021_260_MOESM1_ESM.pdf]

Associations between the built environment and obesity - An umbrella review

Manuscript submitted to International Journal of Health Geographics

Thao Minh Lam*, Ilonca Vaartjes, Diederick E. Grobbee, Derek Karssenberg and Jeroen Lakerveld

*Corresponding author: Thao Lam, Email: t.m.lam@amsterdamumc.nl

SUPPLEMENTARY MATERIALS

Appendix 1. PRISMA checklist for reporting

Section/topic	#	Checklist item	Reported on page #
**TITLE**			
Title	1	Identify the report as a systematic review, meta-analysis, or both.	1
**ABSTRACT**			
Structured summary	2	Provide a structured summary including, as applicable: background; objectives; data sources; study eligibility criteria, participants, and interventions; study appraisal and synthesis methods; results; limitations; conclusions and implications of key findings; systematic review registration number.	2
**INTRODUCTION**			
Rationale	3	Describe the rationale for the review in the context of what is already known.	3,4
Objectives	4	Provide an explicit statement of questions being addressed with reference to participants, interventions, comparisons, outcomes, and study design (PICOS).	3,4
**METHODS**			
Protocol and registration	5	Indicate if a review protocol exists, if and where it can be accessed (e.g., Web address), and, if available, provide registration information including registration number.	4
Eligibility criteria	6	Specify study characteristics (e.g., PICOS, length of follow-up) and report characteristics (e.g., years considered, language, publication status) used as criteria for eligibility, giving rationale.	4
Information sources	7	Describe all information sources (e.g., databases with dates of coverage, contact with study authors to identify additional studies) in the search and date last searched.	4
Search	8	Present full electronic search strategy for at least one database, including any limits used, such that it could be repeated.	Appendix 1
Study selection	9	State the process for selecting studies (i.e., screening, eligibility, included in systematic review, and, if applicable, included in the meta-analysis).	4
Data collection process	10	Describe method of data extraction from reports (e.g., piloted forms, independently, in duplicate) and any processes for obtaining and confirming data from investigators.	5
Data items	11	List and define all variables for which data were sought (e.g., PICOS, funding sources) and any assumptions and simplifications made.	5

Risk of bias in individual studies	12	Describe methods used for assessing risk of bias of individual studies (including specification of whether this was done at the study or outcome level), and how this information is to be used in any data synthesis.	5
Summary measures	13	State the principal summary measures (e.g., risk ratio, difference in means).	5
Synthesis of results	14	Describe the methods of handling data and combining results of studies, if done, including measures of consistency (e.g., I^2) for each meta-analysis.	5

Section/topic	#	Checklist item	Reported on page #
Risk of bias across studies	15	Specify any assessment of risk of bias that may affect the cumulative evidence (e.g., publication bias, selective reporting within studies).	5
Additional analyses	16	Describe methods of additional analyses (e.g., sensitivity or subgroup analyses, meta-regression), if done, indicating which were pre-specified.	-
**RESULTS**			
Study selection	17	Give numbers of studies screened, assessed for eligibility, and included in the review, with reasons for exclusions at each stage, ideally with a flow diagram.	5, 6 & Figure 1
Study characteristics	18	For each study, present characteristics for which data were extracted (e.g., study size, PICOS, follow-up period) and provide the citations.	Table 1
Risk of bias within studies	19	Present data on risk of bias of each study and, if available, any outcome level assessment (see item 12).	Appendix 4
Results of individual studies	20	For all outcomes considered (benefits or harms), present, for each study: (a) simple summary data for each intervention group (b) effect estimates and confidence intervals, ideally with a forest plot.	Table 1
Synthesis of results	21	Present results of each meta-analysis done, including confidence intervals and measures of consistency.	-
Risk of bias across studies	22	Present results of any assessment of risk of bias across studies (see Item 15).	-
Additional analysis	23	Give results of additional analyses, if done (e.g., sensitivity or subgroup analyses, meta-regression [see Item 16]).	-
**DISCUSSION**			
Summary of evidence	24	Summarize the main findings including the strength of evidence for each main outcome; consider their relevance to key groups (e.g., healthcare providers, users, and policy makers).	11 - 13 + Table 2
Limitations	25	Discuss limitations at study and outcome level (e.g., risk of bias), and at review-level (e.g., incomplete retrieval of identified research, reporting bias).	15
Conclusions	26	Provide a general interpretation of the results in the context of other evidence, and implications for future research.	16
**FUNDING**			
Funding	27	Describe sources of funding for the systematic review and other support (e.g., supply of data); role of funders for the systematic review.	-

For more information, visit: **www.prisma-statement.org**.

Appendix 2. Full search strategy for all databases

A1.1 MEDLINE/ PubMed

Aspect	PubMed search string
Aspect 1: Outcome	"Body Weights and Measures"[Mesh] OR "body composition"[Mesh] OR "abdominal fat"[Mesh] OR obesit*[tiab] OR obese[tiab] OR body weight[tiab] OR overweight[tiab] OR BMI[tiab] OR body mass[tiab] OR body fat[tiab] OR body composition[tiab] OR body shape[tiab] OR adiposity[tiab] OR skinfold*[tiab] OR weight status[tiab] OR abdominal fat[tiab] OR "Overnutrition"[Mesh] NOT "Pediatric Obesity"[Mesh]
Aspect 2: Study type	(meta[Title] OR review[Title] OR ("Review"[Publication Type] OR "Meta-Analysis as Topic"[Mesh] OR "Review Literature as Topic"[Mesh] OR "Meta-Analysis "[Publication Type] OR "Systematic Review "[Publication Type]))
Aspect 3: Exposure	"Residence Characteristics"[Majr:NoExp] OR "Social Planning"[Mesh] OR "Fast Foods"[Mesh] OR "Food Supply"[Mesh] OR "Environment"[Mesh] OR "Air Pollution"[Mesh:NoExp] OR "built environment"[Mesh] OR "Urbanization"[Mesh] OR "Geographic Information Systems"[Mesh] OR "physical environment"[tiab] OR "physical activity environment"[tiab] OR “physical activity”[tiab] OR recreational facilit*[tiab] OR sports facilit*[tiab] OR “park”[tiab] OR “parks”[tiab] OR “parking”[tiab] OR hiking trail*[tiab] OR walking trail*[tiab] OR "green space"[tiab] OR bike path*[tiab] OR cycle path*[tiab] OR recreational acivit*[tiab] OR activity center*[tiab] OR activity centre*[tiab] OR "open space"[tiab] OR active transport*[tiab] OR private transport*[tiab] OR passive transport*[tiab] OR greenery[tiab] OR greenness[tiab] OR walkab*[tiab] OR sedentary behavio*[tiab] OR public transport*[tiab] OR "land use"[tiab] OR street connectivity[tiab] OR "land use mix"[tiab] OR "urban sprawl"[tiab] OR "urban design"[tiab] OR "community design"[tiab] OR urban form*[tiab] OR "traffic"[tiab] OR land development*[tiab] OR "spatial planning"[tiab] OR "proximity"[tiab] OR "food environment"[tiab] OR "retail food environment"[tiab] OR "nutrition environment"[tiab] OR food retail*[tiab] OR food access*[tiab] OR fast food*[tiab] OR food resource*[tiab] OR food source*[tiab] OR supermarket*[tiab] OR restaurant*[tiab]
Aspect 4: Geographical range	rural*[tiab] OR urban*[tiab] OR local*[tiab] OR district*[tiab] OR communit*[tiab] OR neighborhood[tiab] OR neighbourhood[tiab]

Search	Query	Items found
#1	Aspect 1	932378
#2	Aspect 2	2759091
#3	Aspect 3	1475743
	#1 AND #2 AND #3	8376

#4	Aspect 4	1968450
#5	#1 AND #2 AND #3 AND #4	1069
#6<br>human filter	#5 NOT (animals[mh] NOT humans[mh])	1013
#7<br>adult filter	#5 NOT (("Adolescent"[Mesh] OR "Child"[Mesh] OR "Infant"[Mesh] OR adolescen*[tiab] OR child*[tiab] OR schoolchild*[tiab] OR infant*[tiab] OR girl*[tiab] OR boy*[tiab] OR teen[tiab] OR teens[tiab] OR teenager*[tiab] OR youth*[tiab] OR pediatr*[tiab] OR paediatr*[tiab] OR puber*[tiab]) NOT ("Adult"[Mesh] OR adult*[tiab] OR man[tiab] OR men[tiab] OR woman[tiab] OR women[tiab]))	865
#8 human adults	#6 NOT (animals[mh] NOT humans[mh])	809
#9 Final string	#8 *+ filters (Year publication (1/1/2000 – 1/6/2019) + English)*	706

A1.2 EMBASE

Aspect	EMBASE search string
Aspect 1:	‘body fat’/exp OR ‘body mass’/exp OR ‘body size’/exp OR ‘body weight’/exp OR ‘weight circumference’/exp OR ‘waist hip ratio’/exp OR ‘weight to height ratio’/exp OR ‘weight height ratio’/exp OR ‘body composition’/exp OR ‘abdominal fat’/exp OR ‘obesit*’:ti,ab,kw OR ‘obese’:ti,ab,kw OR ‘body weight’:ti,ab,kw OR ‘overweight’:ti,ab,kw OR ‘BMI’:ti,ab,kw OR ‘body mass’:ti,ab,kw OR ‘body fat’:ti,ab,kw OR ‘body composition’:ti,ab,kw OR ‘body shape’:ti,ab,kw OR ‘adiposity’:ti,ab,kw OR ‘skinfold*’:ti,ab,kw OR ‘weight status’:ti,ab,kw OR ‘abdominal fat’:ti,ab,kw OR ‘overnutrition’/exp NOT (‘childhood obesity’/exp OR ‘maternal obesity’/exp)
Aspect 2:	‘meta’:ti OR ‘review’:ti OR ‘meta analysis’/exp OR ‘systematic review’/exp OR ‘review’/exp OR ‘systematic review*’:ti,ab,kw OR ‘literature review*’:ti,ab,kw OR ‘meta analys*’:ti,ab,kw OR ‘review of reviews’:ti,ab,kw OR ‘umbrella review*’:ti,ab,kw OR ‘pooled analys*’:ti,ab,kw OR ‘overview*’:ti,ab,kw
Aspect 3:	‘environmental factor’/exp OR ‘environmental exposure’ OR ‘environmental planning’/exp OR ‘fast food’/exp OR ‘food availability’/exp OR ‘land use’/exp OR ‘air pollution’/exp OR ‘light pollution’/exp OR ‘noise pollution’/exp OR ‘traffic pollution’/exp OR ‘urbanization’/exp OR ‘geographic information systems’/exp OR ‘physical activity, capacity and performance’/exp OR ‘diet’/exp OR ‘recreation’ OR ‘sport’/exp OR ‘bicycle’/exp OR ‘motor vehicle’/exp OR ‘traffic’/exp OR ‘sedentary lifestyle’/exp OR ‘catering service’/exp OR ‘physical environment’:ti,ab,kw OR ‘physical activity environment’:ti,ab,kw OR ‘physical activity’:ti,ab,kw OR ‘recreational facilit*’:ti,ab,kw OR ‘sports facilit*’:ti,ab,kw OR ‘park’:ti,ab,kw OR ‘parks’:ti,ab,kw OR ‘green space’:ti,ab,kw OR ‘greenspace’:ti,ab,kw OR ‘recreational acivit*’:ti,ab,kw OR ‘open space’:ti,ab,kw OR ‘active transport*’:ti,ab,kw OR ‘greenery’:ti,ab,kw OR ‘greenness’:ti,ab,kw OR ‘walkab*’:ti,ab,kw OR ‘sedentary behavio*’:ti,ab,kw OR ‘public

	transport*’:ti,ab,kw OR ‘land use’:ti,ab,kw OR ‘street connectivity’:ti,ab,kw OR ‘land use mix’:ti,ab,kw OR ‘urban sprawl’:ti,ab,kw OR ‘urban design’:ti,ab,kw OR ‘community design’:ti,ab,kw OR ‘urban form*’:ti,ab,kw OR ‘traffic’:ti,ab,kw OR ‘land development*’:ti,ab,kw OR ‘spatial planning’:ti,ab,kw OR ‘proximity’:ti,ab,kw OR ‘food environment’:ti,ab,kw OR ‘retail food environment’:ti,ab,kw OR ‘nutrition environment’:ti,ab,kw OR ‘food retail*’:ti,ab,kw OR ‘food access*’:ti,ab,kw OR ‘fast food*’:ti,ab,kw OR ‘food resource*’:ti,ab,kw OR ‘food source*’:ti,ab,kw OR ‘supermarket*’:ti,ab,kw OR ‘restaurant*’:ti,ab,kw
Aspect 4	‘rural*’:ti,ab,kw OR ‘urban*’:ti,ab,kw OR ‘district*’:ti,ab,kw OR ‘neighborhood’:ti,ab,kw OR ‘neighbourhood’:ti,ab,kw
Final string	#1 AND #2 AND #3 AND #4 NOT ([animals]/lim NOT [humans]/lim) NOT (('adolescent'/exp OR 'child'/exp OR adolescent*:ti,ab OR child*:ti,ab OR schoolchild*:ti,ab OR infant*:ti,ab OR girl*:ti,ab OR boy*:ti,ab OR teen:ti,ab OR teens:ti,ab OR teenager*:ti,ab OR youth*:ti,ab OR pediatr*:ti,ab OR paediatr*:ti,ab OR puber*:ti,ab) NOT ('adult'/exp OR 'aged'/exp OR 'middle aged'/exp OR adult*:ti,ab OR man:ti,ab OR men:ti,ab OR woman:ti,ab OR women:ti,ab))<br><br>*+ Year >1999 + English language filters*

A1.3 CINAHL

Aspect 1:	MH "Body Weights and Measures" OR "Body Composition" OR "Adipose Tissue” OR “Obesity” NOT “Pediatric Obesity”<br><br>AB "weight circumference" OR "body composition" OR "abdominal fat" OR obesit* OR "obese" OR "body weight" OR "overweight" OR "BMI" OR "body mass" OR "body mass index" OR "body fat" OR "body shape" OR "adiposity" OR skinfold* OR "weight status" OR "abdominal fat" OR "waist-hip ratio" OR "waist hip ratio" OR "overnutrition" NOT “childhood obesity”
Aspect 2:	MH “Literature Review” OR “Meta Analysis”<br><br>AB “meta” OR “review” OR “pooled analysis” OR “overview”
Aspect 3:	MH “Communities” OR “Geographic Factors” OR ”Public Spaces” OR “Fast Foods” OR “Obesogenic Environment” OR “Built Environment” OR “Air Pollution” OR "Urban Health" OR "Geographic Information Systems" OR “Environment and Public Health” “Physical activity”<br><br>AB "obesogen" OR "built environment" OR "fast food" OR "air pollution" OR "geographic information system" OR "physical environment" OR "physical activity" OR "food environment" OR "urbanization" OR "green space" OR greenness OR walkability OR sedentary OR "public transport" OR "land use" OR "urban sprawl" OR "urban design" OR "community design" OR "urban form" OR "traffic" OR "spatial planning" OR

	"proximity" OR food retail OR food access OR supermarket OR restaurant
Aspect 4	AB rural OR urban OR surrounding OR local OR district OR region OR community OR neighborhood OR neighbourhood

Final string: (MH ("Body Weights and Measures" OR "Body Composition" OR "Adipose Tissue" OR "Obesity") OR AB ("weight circumference" OR "body composition" OR "abdominal fat" OR obesit* OR "obese" OR "body weight" OR "overweight" OR "BMI" OR "body mass" OR "body mass index" OR "body fat" OR "body shape" OR "adiposity" OR skinfold* OR "weight status" OR "abdominal fat" OR "waist-hip ratio" OR "waist hip ratio" OR "overnutrition" NOT "childhood obesity")) AND (MH ("Literature Review" OR "Meta Analysis") OR AB ("meta" OR "review" OR "pooled analysis" OR "overview")) AND (MH ("Communities" OR "Geographic Factors" OR "Public Spaces" OR "Fast Foods" OR "Obesogenic Environment" OR "Built Environment" OR "Air Pollution" OR "Urban Health" OR "Geographic Information Systems" OR "Environment and Public Health" "Physical activity") OR (AB ("obesogen" OR "built environment" OR "fast food" OR "air pollution" OR "geographic information system" OR "physical environment" OR "physical activity" OR "food environment" OR "urbanization" OR "green space" OR greenness OR walkability OR sedentary OR "public transport" OR "land use" OR "urban sprawl" OR "urban design" OR "community design" OR "urban form" OR "traffic" OR "spatial planning" OR "proximity" OR food retail OR food access OR supermarket OR restaurant))) AND (AB (rural OR urban OR surrounding OR local OR district OR region OR community OR neighborhood OR neighbourhood)) *+ English language & publication 2000-2019 filters*

A1. 4 SCOPUS

Final string: *TITLE-ABS-KEY ("obesogen" OR "built environment" OR "fast food" OR "air pollution" OR "geographic information system" OR "physical environment" OR "physical activity" OR "food environment" OR "urbanization" OR "green space" OR greenness OR walkability OR sedentary OR "public transport" OR "land use" OR "urban sprawl" OR "urban design" OR "community design" OR "urban form" OR "traffic" OR "spatial planning" OR "proximity" OR food AND retail OR food AND access OR supermarket OR restaurant) AND TITLE-ABS-KEY (review OR meta OR overview OR "pooled analysis") AND LANGUAGE (english) AND PUBYEAR > 1999 AND TITLE-ABS-KEY (obesity OR obese OR "overweight" OR "BMI" OR "body mass index" OR "body fat" OR adiposity OR skinfold OR "weight status" AND NOT ("childhood obesity" OR "pediatric obesity"))*

A1.5 PROSPERO

Final string: environment AND obes*

A1.6 JBI

Final string: ("fast food" OR "environment" OR "geographic information system") AND ("weight circumference" OR "body composition" OR "abdominal fat" OR obesit* OR "obese" OR "body weight" OR "overweight" OR "BMI")

A1.7 CDSR

Final string: environment AND obes*

Appendix 3. Full references of included reviews

16. Allender S, Foster C, Hutchinson L, Arambepola C. Quantification of urbanization in relation to chronic diseases in developing countries: A systematic review. J Urban Heal. 2008;85:938–51.

17. Lachowycz K, Jones AP. Greenspace and obesity: A systematic review of the evidence. Obes Rev. 2011;12:183–9.

18. Grasser G, Van Dyck D, Titze S, Stronegger W. Objectively measured walkability and active transport and weight-related outcomes in adults: a systematic review. Int J Public Health [Internet]. G. Grasser, Institute of Social Medicine and Epidemiology, Medical University of Graz, Graz, Austria., Switzerland; 2013;58:615–25. Available from: http://www.embase.com/search/results?subaction=viewrecord&from=export&id=L563045604

19. Papas MA, Alberg AJ, Ewing R, Helzlsouer KJ, Gary TL, Klassen AC. The built environment and obesity. Epidemiol Rev. United States; 2007;29:129–43.

20. Cobb LK, Appel LJ, Manuel Franco M, Jones-Smith JC, Alana Nur A, Anderson CA. Systematic Review of Methods , Study Quality and Results. Obes (Silver Spring). 2015;23:1331–44.

21. Sugiyama T, Koohsari MJ, Mavoa S, Owen N. Activity-Friendly Built Environment Attributes and Adult Adiposity. Curr Obes Rep. 2014;3:183–98.

22. Mackenbach JD, Rutter H, Compernolle S, Glonti K, Oppert J-M, Charreire H, et al. Obesogenic environments: a systematic review of the association between the physical environment and adult weight status, the SPOTLIGHT project. BMC Public Health [Internet]. England; 2014;14:233. Available from: http://www.embase.com/search/results?subaction=viewrecord&from=export&id=L605806669

27. Chandrabose M, Rachele JN, Gunn L, Kavanagh A, Owen N, Turrell G, et al. Built environment and cardio-metabolic health: systematic review and meta-analysis of longitudinal studies. Obes Rev [Internet]. M. Chandrabose, Mary MacKillop Institute for Health Research, Australian Catholic University, Melbourne, VIC, Australia, England; 2019;20:41–54. Available from: http://www.embase.com/search/results?subaction=viewrecord&from=export&id=L624078773

28. Schüle SA, Bolte G. Interactive and independent associations between the socioeconomic and objective built environment on the neighbourhood level and individual health: A systematic review of multilevel studies. PLoS One [Internet]. 2015;10. Available from: http://dx.doi.org/10.1371/journal.pone.0123456

29. Black JL, Macinko J. Neighborhoods and obesity. Nutr Rev [Internet]. J. L. Black, New York University, Steinhardt School of Culture, Education, and Human Development, Department of Nutrition, Food Studies and

Public Health, 35 West 4th St., New York, NY 10012, United States, United States; 2008;66:2–20. Available from: http://www.embase.com/search/results?subaction=viewrecord&from=export&id=L351666657

30. Leal C, Chaix B. The influence of geographic life environments on cardiometabolic risk factors: A systematic review, a methodological assessment and a research agenda. Obes Rev. 2011;12:217–30.

31. Feng J, Glass TA, Curriero FC, Stewart WF, Schwartz BS. The built environment and obesity: A systematic review of the epidemiologic evidence. Heal Place [Internet]. Elsevier; 2010;16:175–90. Available from: http://dx.doi.org/10.1016/j.healthplace.2009.09.008

32. Angkurawaranon C, Jiraporncharoen W, Chenthanakij B, Doyle P, Nitsch D. Urban environments and obesity in southeast asia: A systematic review, meta-analysis and Meta-regression. PLoS One. 2014;9:1–19.

33. Hernández A V., Pasupuleti V, Deshpande A, Bernabé-Ortiz A, Miranda JJ. Effect of rural-to-urban within-country migration on cardiovascular risk factors in low- and middle-income countries: A systematic review. Heart. 2012;98:185–94.

34. Wilkins E, Radley D, Morris M, Hobbs M, Christensen A, Marwa WL, et al. A systematic review employing the GeoFERN framework to examine methods, reporting quality and associations between the retail food environment and obesity. Health Place. England; 2019;57:186–99.

35. Holsten JE. Obesity and the community food environment: A systematic review. Public Health Nutr. 2009;12:397–405.

36. Fraser LK, Edwards KL, Cade J, Clarke GP. The geography of fast food outlets: A review. Int J Environ Res Public Health [Internet]. School of Geography, University of Leeds, LS2 9JT, United Kingdom; 2010;7:2290–308. Available from: https://www.scopus.com/inward/record.uri?eid=2-s2.0-77954790272&doi=10.3390%2Fijerph7052290&partnerID=40&md5=2aed319e8ff66222d91c56d9c84adef0

37. Gamba RJ, Schuchter J, Rutt C, Seto EYW. Measuring the food environment and its effects on obesity in the United States: a systematic review of methods and results. J Community Health. Netherlands; 2015;40:464–75.

38. Fleischhacker SE, Evenson KR, Rodriguez DA, Ammerman AS. A systematic review of fast food access studies. Obes Rev. 2011;12:460–71.

39. Giskes K, van Lenthe F, Avendano-Pabon M, Brug J. A systematic review of environmental factors and obesogenic dietary intakes among adults: Are we getting closer to understanding obesogenic environments? Obes Rev [Internet]. School of Public Health, Queensland University of Technology, Kelvin Grove, QLD, Australia; 2011;12:e95–106. Available from: https://www.scopus.com/inward/record.uri?eid=2-s2.0-79954997165&doi=10.1111%2Fj.1467-789X.2010.00769.x&partnerID=40&md5=87ddcadc2181d3c81a9361edccb1cb62

40. Tseng E, Zhang A, Shogbesan O, Gudzune KA, Wilson RF, Kharrazi H, et al. Effectiveness of Policies and Programs to Combat Adult Obesity: a Systematic Review. J Gen Intern Med [Internet]. Journal of General Internal Medicine; 2018;33:1990–2001. Available from: http://dx.doi.org/10.1007/s11606-018-4619-z

41. McCormack GR, Cabaj J, Orpana H, Lukic R, Blackstaffe A, Goopy S, et al. A scoping review on the relations between urban form and health: a focus on Canadian quantitative evidence. Heal Promot Chronic Dis Prev Canada. 2019;39:187–200.

43. Malambo P, Kengne AP, De Villiers A, Lambert E V, Puoane T. Built Environment, Selected Risk Factors and Major Cardiovascular Disease Outcomes: A Systematic Review. PLoS One. United States; 2016;11:e0166846.

44. Renalds A, Smith TH, Hale PJ. A systematic review of built environment and health. Fam Community Heal. 2010;33:68–78.

45. Kondo MC, Fluehr JM, McKeon T, Branas CC. Urban Green Space and Its Impact on Human Health. Int J Environ Res Public Health. Switzerland; 2018;15.

46. Patterson R, Webb E, Hone T, Millett C, Laverty AA. Associations of Public Transportation Use with Cardiometabolic Health: A Systematic Review and Meta-Analysis. Am J Epidemiol. 2019;188:785–95.

47. An R, Ji M, Yan H, Guan C, R. A, M. J, et al. Impact of ambient air pollution on obesity: A systematic review. Int J Obes [Internet]. R. An, Department of Kinesiology and Community Health, University of Illinois at Urbana-Champaign, Champaign, IL, United States, England; 2018;42:1112–26. Available from: http://www.embase.com/search/results?subaction=viewrecord&from=export&id=L622282764

48. Casagrande SS, Whitt-Glover MC, Lancaster KJ, Odoms-Young AM, Gary TL. Built Environment and Health Behaviors Among African Americans. A Systematic Review. Am J Prev Med [Internet]. American Journal of Preventive Medicine; 2009;36:174–81. Available from: http://dx.doi.org/10.1016/j.amepre.2008.09.037

49. Lovasi GS, Hutson MA, Guerra M, Neckerman KM, G.S. L, M.A. H, et al. Built environments and obesity in disadvantaged populations. Epidemiol Rev. G. S. Lovasi, Robert Wood Johnson Foundation Health, Society Scholars Program, Institute of Social and Economic Research and Policy, Columbia University, 420 West 118th Street, New York, NY 10027, United States; 2009;31:7–20.

50. Larson NI, Story MT, Nelson MC. Neighborhood environments: disparities in access to healthy foods in the U.S. Am J Prev Med. Netherlands; 2009;36:74–81.

51. Ferdinand AO, Sen B, Rahurkar S, Engler S, Menachemi N. The Relationship Between Built Environments and Physical Activity: A Systematic Review. Am J Public Health [Internet]. Department of Health Care Organization and Policy, University of Alabama at Birmingham.: American Public Health Association; 2012;102:e7–13. Available from: http://search.ebscohost.com/login.aspx?direct=true&db=cin20&AN=104421912&site=ehost-live

59. Durand CP, Andalib M, Dunton GF, Wolch J, Pentz MA. A systematic review of built environment factors related to physical activity and obesity risk: Implications for smart growth urban planning. Obes Rev [Internet]. C.P. Durand, Institute for Health Promotion and Disease Prevention Research, Department of Preventive Medicine, University of Southern California, 1000 South Fremont Avenue, Unit 8 Bldg 5, Alhambra, CA 91803, United States, England; 2011;12:e173–82. Available from: http://www.embase.com/search/results?subaction=viewrecord&from=export&id=L51358616

Appendix 4. Risk of bias assessment of included studies using ROBIS tool

First author + year of publication	Concerns regarding specification of study eligibility criteria	Concerns regarding methods used to identify and/or select studies	Concerns regarding methods used to collect data and appraise studies	Concerns regarding methods used to synthesize results	Risk of bias in the review
Allender 2008	Unclear	Low	Unclear	Low	Unclear
An 2018	Unclear	Unclear	Unclear	Unclear	Unclear
Angkurawaranon 2014	Unclear	Low	Unclear	Low	Low
Black Macinko 2008	Unclear	Unclear	High	Unclear	Unclear
Casagrande 2009	Unclear	High	High	Low	Unclear
Chandrabose 2019	Unclear	Low	Low	Low	Low
Cobb 2015	Unclear	Unclear	Low	Low	Unclear
Durand 2011	Unclear	High	High	Unclear	High
Feng 2009	Low	Unclear	High	Low	Unclear
Ferdinand 2012	Low	Low	High	Unclear	Unclear
Fleischhacker 2011	Unclear	Unclear	High	Unclear	Unclear
Fraser 2010	Unclear	Unclear	High	Unclear	Unclear
Gamba 2015	Unclear	High	High	Unclear	High
Giske 2011	Unclear	Unclear	High	Unclear	Unclear
Grasser 2013	Low	Low	Unclear	Low	Unclear
Hernandez 2012	Low	Low	Unclear	Low	Low
Holsten 2008	Unclear	Unclear	High	High	High
Kondo 2018	Unclear	Unclear	High	Unclear	Unclear
Lachowycz 2011	Unclear	Unclear	Low	Unclear	Unclear
Larson 2009	Unclear	High	High	Unclear	Unclear
Leal and Chaix 2010	Unclear	Unclear	Unclear	Unclear	Unclear
Lovasi 2009	Low	Unclear	High	Unclear	Unclear
Mackenbach 2014	Unclear	Low	Low	Low	Low
Malambo 2016	Unclear	High	Low	High	High
McCormack 2019	Low	Unclear	High	High	High
Papas 2007	Unclear	Unclear	High	Unclear	Unclear
Patterson 2019	Low	Low	Unclear	Low	Low
Renalds 2010	High	High	High	High	High
Schule 2015	Unclear	Unclear	High	High	High
Sugiyama 2014	Unclear	Unclear	High	Unclear	Unclear
Tseng 2018	Unclear	Low	Low	Low	Low
Wilkins 2019	Low	Unclear	Low	Low	Low
